# Upstream factors impacting COVID-19 vaccination rates across Africa: A systematic review protocol

**DOI:** 10.1371/journal.pone.0310884

**Published:** 2024-09-25

**Authors:** Obidimma Ezezika, Tiana Stephanie Kotsaftis, Alanna Marson

**Affiliations:** 1 Faculty of Health Sciences, Global Health & Innovation Lab, School of Health Studies, University of Western Ontario, London, Canada; 2 African Centre for Innovation & Leadership Development, Abuja, Nigeria; 3 Western Libraries, Western University, London, London, Canada; National center for chronic and non-communicable diesease prevention and control, CHINA

## Abstract

**Background:**

Upstream factors have been found to affect COVID-19 vaccination rates and coverage globally. However, there are inadequate details within the African context. This systematic review aims to close this research gap by investigating upstream factors influencing COVID-19 vaccination rates in Africa.

**Methods:**

A literature search will be systematically conducted utilizing various databases including: MEDLINE, EMBASE, SCOPUS, CINAHL, Web of Science, and PsycINFO. Eligible studies will include peer-reviewed articles published in the English language from 2020–2023, conducted in Africa, focused on upstream factors, and include one barrier or facilitator to COVID-19 vaccination rates. Two reviewers will use a two-step screening process to examine every article’s title, abstract, and full text. A third-party reviewer will resolve disagreements between both individual reviewers. This review will focus on extracting data from published studies to explain the upstream factors included and their impact on COVID-19 vaccination rates across Africa. Data and records will be managed using Covidence. Preferred Reporting Items for Systematic Reviews and Meta-Analyses [PRISMA] framework will be used as the basis for reporting. To reduce bias, the researchers will use the Mixed Methods Appraisal Tool to assess the studies chosen for review. Results will be compiled utilizing four tables to summarize articles and group determinants based on the Consolidated Framework for Implementation Research (CFIR).

**Discussion:**

Upstream factors have been cited as affecting population health, vaccination programs, and COVID-19, yet a large-scale systematic review has not been conducted to investigate these factors in relation to COVID-19 vaccination disparities faced in Africa. This review aims to analyze the root causes of African vaccination disparities by focusing on upstream factors. Understanding these factors is vital to help explain why these disparities occur and for designing effective interventions for future vaccinations. The results are expected to provide insights for researchers, policymakers, health systems, and individuals by identifying how resources and efforts can be better utilized to improve vaccination uptake and access.

**Trial registration:**

Systematic review registration: CRD42024501293.

## Background

By the beginning of 2022, less than 10% of Africa had been fully vaccinated for COVID-19. In contrast, half of the world was fully vaccinated at this time–countries like Canada and the United States had 77% and 63% of their population vaccinated [1] By mid-2022, only four Sub-Saharan African countries had met their 70% vaccination target [2]. As of March 2023, Seychelles had the highest vaccination rate on the continent, with a value of 204.87, while Burundi had the lowest, with a value of 0.24 [3]. The variation in vaccination coverage has previously been established within the region. A study focused on distribution and determinants of COVID-19 vaccination from March 2021 to June 2022 concluded that even with vaccine coverage increase, inequality remains with total cases and the human development index (HDI) serving as significant factors of COVID-19 vaccination [4]. Another review considered steps African countries were taking to reduce these gaps; however, it focused only on vaccine supply, partnerships, vaccine sourcing, and other coordinated regional efforts, from an organizational and governmental perspective [5]. Several reviews and studies on COVID-19 vaccination rates in Africa have focused on hesitancy and uptake to the exclusion of upstream determinants that might explain issues in vaccine scale-up and disparities between countries [6–9]. This review seeks to fill this research gap by analyzing upstream factors that serve as barriers and facilitators to COVID-19 vaccination across Africa.

A review considering the unequal distribution of COVID-19 vaccines in Africa referred to the COVID-19 pandemic as a “*syndemic pandemic”* highlighting that COVID interacts with social determinants of health, thus exacerbating these disparities [10].

Recognizing the need to investigate these disparities from a holistic, root-cause perspective, we focused on upstream factors to help explain these disparities. Upstream factors refer to foundational elements that influence health outcomes. This includes factors that fall within the jurisdiction of governments (national and local), international organizations, and non-governmental organizations [11]. Various previous literature has considered upstream factors and their role in creating and reducing health inequities. A broad study conducted in the United States considered the effect of upstream factors on improving health and reducing disparities [12], concluding that neighborhood conditions, additional income and health, childhood education, and civil rights movements may all have an impact on health and disparities [8]. More narrow studies have also considered upstream factors, such as the relationship between upstream factors and oral health [13].

Upstream factors have previously been explored in relation to other vaccination programs to help explain barriers and facilitators. A study with broad country considerations, including African countries, cited upstream factors such as urban vs rural place of residence, education, financial factors, and availability of healthcare services as social determinants that affect child immunization [14]. In another study, child immunization was inversely related to upstream factors such as urban residence, maternal education, and affordability in India [15]. Another study considered factors that influence HPV vaccination among immigrant parents, citing financial issues, immigration laws, and housing conditions as key factors that influence HPV vaccination rates [16].

Africa WHO highlighted a list of challenges in Africa that have impacted the implementation of the COVID-19 program [17]. Factors include lack of funds, lack of professionals and insufficient funding [17]. These factors relate to upstream factors, which have been explored further in reviews and individual studies. In Nigeria, the high level of COVID-19 vaccination disparity within the country was explored, which revealed that place of residence was a key determinant impacting the inequity and called for a holistic, integrated policy approach along with vaccine education [18]. A scoping review with similar aims to this systematic review focused on individual, interpersonal, and structural barriers/facilitators to COVID-19 vaccine uptake but did not address access and its relation to social determinants of health [15]. It mentioned inaccessibility and perceived information as a barrier which can be further explored [19]. Another study on low- and middle-income countries focused on COVID-19 vaccination hesitancy and uptake by analyzing upstream factors, including education and trust [20]. Furthermore, a study focused on only low-income countries considered socioeconomic status, education, occupation, and geographical landscape as plausible upstream factors that may inhibit access to COVID-19 vaccinations in these countries, thus creating inequities [21]. Although research has begun to consider upstream factors for COVID-19 vaccination in Africa, there is a paucity of synthesis of these upstream factors.

Outside of the African context, determinants of COVID-19 vaccination have been explored. A study conducted in the United States considered individual, communication and social determinants associated with uptake and concluded that race/ethnicity, risk perception and media are factors that affect COVID-19 vaccination uptake [22]. Health inequalities, systemic racism and socioeconomic disadvantages have also been cited as factors related to impacting COVID-19 vaccination in relation to hesitancy on a worldwide level through a scoping review conducted [23]. An additional review in Europe concluded that factors including low literacy, insecure housing, staff shortages and absence of policies and promotion were cited as key factors inhibiting vaccine access for migrants [24].

Upstream factors and their established impact on population health sets the stage for this relationship to be considered to help explain COVID-19 vaccination disparities in Africa. Despite research laying this foundation, a systematic review exploring upstream factors and their impact on disparities of COVID-19 vaccination in Africa has not been conducted. This systematic review aims to fill this research gap and explain upstream factors that impact COVID-19 vaccination disparities in Africa for future COVID-19 vaccination programs and existing and future vaccination programs.

## Methods

### Protocol registration and reporting

The registration number of this protocol in PROSPERO is CRD42024501293. The Preferred Reporting Items for Systematic Reviews and Meta-Analyses Protocols (PRISMA-P) statement was utilized to guide the reporting of this protocol (S1 Data).

### Information sources and search strategy

#### Eligibility criteria

Study inclusion criteria.

*Population*. This systematic review will include all individuals as long as they live in an African country and are included in various statistics/indicators.

*Intervention/Exposure*. No specific intervention or exposure must be included in studies as long as it considers COVID-19 vaccination and its uptake or rates.

*Comparator*. No comparison group or intervention for this review.

*Outcomes of interest*. Studies will be included if they: (1) discuss upstream factors impacting COVID-19 Vaccination Rates Across Africa.

Upstream factors refer to foundational elements that determine whether individuals or communities can access essential services needed to improve health outcomes [25]. These factors include items controlled by local and national governments, international and non-governmental organizations [11].They include the infrastructure and capabilities of health systems, as well as the policies and management practices that affect access to healthcare resources like medical supplies [11]. Upstream factors also encompass community traits such as the built environment layout and infrastructure, which impact access. Any characteristic that affects resource distribution or is controlled by policy is considered an upstream factor. Examples of upstream factors are included in Table 1.

**Table 1:** Examples of upstream factors.

**Vaccine Supply**	*The number of COVID-19 vaccines available for purchase or for use of countries*
**Manufacturing capacity of countries**	*The ability of individual countries to produce vaccines within*
**Policy/Laws**	*Regulations and guidelines established by various levels of government to guide vaccine approval and distribution*
**Procurement**	*Avenues of obtaining vaccines through purchase*
**Delivery/transportation of vaccines**	*Process of COVID-19 vaccinations reaching their target destination along with storage capabilities*
**Resource Allocation**	*Management of COVID-19 vaccines in each country impacting the availability*, *distribution*, *and administration of vaccines*. *Effective resource management ensures timely and equitable access*
**COVAX distribution**	*Actions*, *allocation and steps taken by COVAX to ensure or guide their donations and dose-sharing*
**Community leadership in vaccine access**	*Role of community members who hold status in acquiring and distributing vaccines*
**Healthcare capacity**	*Ability of healthcare systems and clinics to provide vaccination services and handle their population*
**Geography/ environment**	*Characteristics and placement of countries and communities including proximity to vaccines and rural vs urban*

Overall, upstream factors focus on access and availability rather than the individual’s decision to take the vaccine. In contrast, downstream factors emphasize personal agency to improve health outcomes [26]. For example, if rural areas have no access to vaccination due to low vaccine rollout, that would be considered an upstream issue. However, if an individual living in a rural area and can’t access the available vaccine due to personal issues related to transportation or funds, that would be considered a downstream factor.

*Setting*. Studies will be included if they are conducted in an African country or conducted in low-middle income countries if they consider at least one African country.

*Study design*. Eligible studies must be peer-reviewed and from an academic journal. Studies may include research data/themes, lived experience from individuals and insights from stakeholders. Studies must be published in the English language, after 2020. This time period was selected because COVID-19 was declared a pandemic in 2020.

### Exclusion criteria

Studies will be excluded based on the following criteria: the study only addresses vaccine hesitancy; the study only focuses on personal/individual factors; articles that are investigating clinical factors of COVID-19 vaccine efficacy or safety; studies that only consider a global context; studies published before 2020 and published in languages other than the English language; studies that are not peer-reviewed; articles that are secondary analyses that do not provide new insights or themes and articles that exclusively focus on downstream factors such as post-vaccine monitoring, vaccine hesitancy/beliefs, vaccine awareness, ondividual’s beliefs and attitudes towards the COVID-19 vaccine, social and economic standing or class of individuals or communities inclusive of income, employment, and education.

### Search strategy

Searches were completed using the following databases: MEDLINE, EMBASE, Scopus, CINAHIL, Web of Science and PsycINFO. Searches were completed, and results were exported on February 7, 2024.

In addition to the database searches, we will perform forward and backward citation searches to find additional articles that meet the selection criteria.

Example search terms include: immunization, vaccination, vaccination hesitancy, Africa, rate, uptake, coverage, barrier, Coronavirus, corona virus, nCov, covid, sarscov, and Africa. Covid-related terms were chosen based on the expert searches developed by Wolters Kluwer to ensure the comprehensiveness of results. Details of the search in MEDLINE can be found in S2 Data.

### Study records

#### Data management

Data and records will be managed using Covidence. Covidence is a web-based software platform that streamlines the production of systematic reviews, including Cochrane Reviews. A project will be set up on Covidence (this project is encrypted and shared with all reviewers). Preferred Reporting Items for Systematic Reviews and Meta-Analyses [PRISMA] framework will be used as the basis for reporting.

#### Selection process

Two reviewers will use a two-step screening process to examine the title, abstract, and full text for every article that made it through the first round. Articles that make it through both rounds of screening will have their references checked for any relevant studies to include. A third-party reviewer will resolve disagreements between individual both reviewers.

This review will focus on extracting data from published studies to explain upstream factors included and their impact on COVID-19 vaccination rates across Africa. This will include country/countries studied, population, methods, upstream factor(s) explored along with a rationale and barriers/facilitators. Two researchers will be responsible for independently extracting and checking collected data. Disagreements between researchers will be resolved by consensus-based discussion or a third-party reviewer.

#### Dealing with missing data

Missing data will be specified in the review, and study investigators will not be contacted for details regarding specific articles.

#### Data synthesis

After the completion of data extraction, the two researchers, along with the corresponding author, will prepare four tables to weave our results. Table one will comprise the characteristics of the included studies, including the author and year, country, study design, participants, study setting, and objective. These characteristics will be extracted and highlighted with the main outcome of comparing studies to be able to extract themes from various locations, different factors and groups. Table two will group together all determinants, including barriers and the facilitators, and how they match to the Consolidated Framework for Implementation Research (CFIR) domains and constructs, specifically the outer setting and process domains [27]. Table three will be a frequency table of the cited CFIR constructs to quantify the number of barriers and facilitators that account for each CFIR construct. Lastly, the barriers and facilitators from Table two will be examined and analyzed. Three team members will hold multiple meetings to create a thematic synthesis table extracted from barriers, facilitators, and identified upstream factors. Reoccurring barriers, facilitators, and upstream factors will be reviewed and grouped with similar content, resulting in themes. Each theme will be named and defined to clarify its scope and significance, paired with detailed descriptions of what the themes reveal. Table four will identify the overarching thematic areas across the barriers and facilitators in order to develop a thematic synthesis of our results to evaluate how these factors vary across various countries. Disagreements will be resolved through a consensus-based discussion.

#### Risk of bias in individual studies

Two researchers will be evaluating the articles independently and will be required to evaluate the article based on a checklist for data extraction. To reduce bias, the researchers will use the Mixed Methods Appraisal Tool to assess the studies chosen for review. This tool includes a checklist for qualitative, quantitative, and mixed-methods studies. This tool assesses the quality of methods used in the study to assess the strength of information and brings to light any possible bias.

## Discussion

Upstream factors affecting COVID-19 vaccination have previously been cited in a variety of settings, inclusive of a wide array of factors. Factors cited include, education, income, family household dynamics, poverty, percievied health inequities, unemployment, vaccine education, vaccine inaccessibility, occupation and work status [19,28–30]. Despite these findings, to our knowledge a large-scale systematic review has not been conducted to investigate upstream factors in relation to COVID-19 vaccination disparities faced in Africa. Research in the African context has mostly confined COVID-19 vaccination matters to hesitancy and uptake concerns only. A research gap regarding upstream factors and vaccination rates in Africa warrants further investigation. This review seeks to close this research gap by identifying upstream factors impacting vaccine uptake and access within Africa. Additionally, this review aims to provide a holistic analysis of the root causes that might contribute to vaccination inequalities in Africa. A table will be compiled to help compare and contrast barriers and facilitators extracted from articles to help summarize upstream factors and thematic insights that impact disparities.

COVID-19 vaccination compared globally and within the continent shows that this inequity is of concern and needs further investigation to help guide vaccination efforts for COVID-19 and other vaccination programs. This review sets itself apart from previous research by considering the established relationship of upstream factors with COVID-19 vaccine disparities. Upstream factors may serve as an avenue to help explain why countries’ vaccination rates vary, and investigating them may help guide future interventions and vaccination programs.

The results are expected to provide insights for researchers, policymakers, health systems and individuals through identifying how resources and efforts can be better utilized to improve vaccination uptake and access. Findings from this review will be shared, allowing the scientific community to learn and build upon the relationship between barriers, facilitators, upstream factors and COVID-19 vaccination uptake. Each barrier and facilitator may be used as guidance to help improve vaccination programs. This may be accomplished by making countries aware of specific areas they can improve on. Although we will highlight themes, it is at each country’s discretion to determine how to integrate recommendations. This may be done by adding to existing programs or implementing a new program that overcomes barriers and strengthens facilitators highlighted in our review.

Overall, this systematic review aims to fill a research gap, highlight the importance of upstream factors and guide vaccination programs to address root causes of COVID-19 vaccination disparities in Africa. Findings may help those within the African continent and other low- and middle-income countries design vaccination programs to better serve populations.

### Limitations

This review will employ the multi-context approach to synthesize data, providing a wide range of evidence allowing cross-setting comparisons among African countries. Although such an approach offers transferable patterns of findings at a continental level, they may be too general to target important local or county characteristics or audiences, leading to the study overlooking the relevance of specific contexts and blurring critical differences across different studies [31]. Additionally, only primary research will be utilized which could result in insights from secondary sources being overlooked.

## Supporting information

S1 Data (DOCX)

S2 Data (DOCX)
