# Supplementary material for: Upstream factors impacting COVID-19 vaccination rates across Africa: A systematic review protocol
Source: PLoS One. 2024 Sep 25;19(9):e0310884. doi: 10.1371/journal.pone.0310884 (PMC11423958; doi:10.1371/journal.pone.0310884)
Supplement: S2 Data — (DOCX) [file pone.0310884.s002.docx]

S2 Data: Search Strings (MEDLINE)

**Database: Ovid MEDLINE(R) ALL <1946 to December 18, 2023>**
**Search Strategy:**
**1**  The challenges to a successful COVID-19 vaccination programme in Africa.m_titl. (1)
**2**  Factors influencing COVID-19 vaccine uptake among adults in Nigeria.m_titl. (1)
**3**  rapid increase in coverage of COVID-19 vaccination, Central African Republic.m_titl. (1)
**4**  (Measuring behavioral and social drivers of COVID-19 vaccination in health workers in Eastern and Southern Africa).m_titl. (2)
**5**  Factors Associated with Limited Vaccine Literacy: Lessons Learnt from Covid-19.m_titl. (1)
**6**  (COVID-19 vaccine hesitancy in rural South Africa: Deepening understanding to increase uptake and access).m_titl. (1)
**7**  Challenges to COVID-19 vaccine introduction in the Democratic Republic of the Congo.m_titl. (1)
**8**  Exploring Challenges to COVID-19 Vaccination in the Darfur Region of Sudan.m_titl. (1)
**9**  (Covid-19 Vaccine Uptake and Associated Factors in Sub-Saharan Africa).m_titl. (1)
**10**  (Qualitative Study Exploring Motivators and Barriers to COVID-19 Vaccine Uptake among Adults in South Africa and Zimbabwe).m_titl. (1)
**11**  (Uptake of COVID-19 vaccines and associated factors among adults in Uganda).m_titl. (1)
**12**  (Pandemic across Africa: Current Status of Vaccinations and Implications for the Future).m_titl. (1)
**13**  Will People in Conflict Affected Zones in Africa Have Access to COVID-19 Vaccine.m_titl. (1)
**14**  (Assessing COVID-19 vaccine hesitancy and barriers to uptake in Sub-Saharan Africa).m_titl. (2)
**15**  Inequality in COVID-19 Vaccination in Africa.m_titl. (1)
**16**  1 or 2 or 3 or 4 or 5 or 6 or 7 or 8 or 9 or 10 or 11 or 12 or 13 or 14 or 15 (17)
**17**  immunization/ or vaccination/ or mass vaccination/ or vaccination coverage/ (160649)
**18**  Anti-Vaccination Movement/ (182)
**19**  vaccination refusal/ or vaccination hesitancy/ (1785)
**20**  ((vaccin* or immuniz* or immunis*) adj3 (rate* or uptake* or success or coverage* or hesitan* or accept* or reject* or reluct* or misconception* or conception* or demand* or literac* or barrier* or driver* or uncertaint* or access* or doses)).tw,kf. (52162)
**21**  17 or 18 or 19 or 20 (188424)
**22**  exp COVID-19 Vaccines/ (24507)
**23**  COVID-19/ (249690)
**24**  SARS-CoV-2/ (163182)
**25**  (coronavirus* or corona virus* or covid* or HCoV* or ncov* or sars-cov* or sarscov*).tw,kf. (409470)
**26**  22 or 23 or 24 or 25 (416172)
**27**  exp Africa/ or (Africa* or Algeria or Angola or Benin or Botswana or "Burkina Faso" or Burundi or "Cabo Verde" or "Cape Verde" or Cameroon or Central African Republic or Chad or Comoros or Congo or "Cote d'Ivoire" or "Ivory Coast" or Djibouti or Egypt or "Equatorial Guinea" or Eritrea or Eswatini or Ethiopia or Gabon or Gambia or Ghana or Guinea or "Guinea-Bissau" or Kenya or Lesotho or Liberia or Libya or Madagascar or Malawi or Mali or Mauritania or Mauritius or Morocco or Mozambique or Namibia or Niger or Nigeria or "Nile Valley" or Rwanda or "Sao Tome" or Principe or Senegal or Seychelles or "Sierra Leone" or Somalia or "South Africa" or "Sub Saharan" or Sudan or Tanzania or Togo or Tunisia or Uganda or Zambia or Zimbabwe).mp. (790135)
**28**  21 and 26 and 27 (1483)
**29**  16 and 28 (16)
